# Hypofractionated short‐course radiotherapy in elderly patients with glioblastoma multiforme: an analysis of the National Cancer Database

**DOI:** 10.1002/cam4.1070

**Published:** 2017-04-24

**Authors:** Kimberley S. Mak, Ankit Agarwal, Muhammad M. Qureshi, Minh Tam Truong

**Affiliations:** ^1^ Boston Medical CenterBoston University School of MedicineBostonMassachusetts

**Keywords:** Elderly, glioblastoma, hypofractionation, radiotherapy, survival

## Abstract

For elderly patients with glioblastoma multiforme (GBM), randomized trials have shown similar survival with hypofractionated short‐course radiotherapy (SCRT) compared to conventionally fractionated long‐course radiotherapy (LCRT). We evaluated the adoption of SCRT along with associated factors and survival in a national patient registry. Using the National Cancer Data Base (NCDB), we identified patients aged ≥70 years with GBM, diagnosed between 1998 and 2011, who received SCRT (34–42 Gy in 2.5–3.4 Gy fractions), or LCRT (58–63 Gy in 1.8–2.0 Gy fractions). Crude and adjusted hazard ratios (HR) were calculated using Cox regression modeling. 4598 patients were identified, 304 (6.6%) in the SCRT group and 4294 (93.4%) in the LCRT group. Median follow‐up was 8.4 months. Median age was 78 versus 75 years, respectively (*P* < 0.0001). Patients who received SCRT had higher Charlson–Deyo comorbidity scores versus LCRT (score of ≥2: 16.9% vs. 10.8%, respectively; *P* = 0.006), and were more likely to be female (53.0% vs. 44.6%, *P* = 0.005). Patients who received SCRT were less likely to undergo chemotherapy (42.8% vs. 79.3%, *P* < 0.0001), more likely to undergo biopsy only (34.5% vs. 19.5%, *P* < 0.0001), and more likely to receive treatment at academic/research programs (49.2% vs. 37.2%, *P* = 0.0001). Median survival was 4.9 months versus 8.9 months, respectively (*P* < 0.0001). The survival detriment with SCRT persisted on multivariable analysis [HR 1.51 (95% CI: 1.33–1.73, *P* < 0.0001)], adjusting for age, gender, race, comorbidities, diagnosis year, facility type, surgery, and chemotherapy. In conclusion, hypofractionated SCRT was associated with worse survival compared to conventionally fractionated LCRT for elderly patients with GBM. Patients who received SCRT were older with worse comorbidities, and were less likely to undergo chemotherapy or resection.

## Introduction

Glioblastoma multiforme (GBM) affects 12,000 people in the United States each year and is the most common primary brain malignancy diagnosed in adults 1, 2, 3. Despite advances in treatment, including the addition of temozolomide (TMZ) to adjuvant radiotherapy (RT), the median overall survival remains dismal at 16–17 months 1, 4, 5, 6, 7. Age remains the most important prognostic factor for patients with GBM, with previous population‐based studies showing an average survival of 4–5 months for patients ≥65 years of age 8, 9, 10. Indeed, GBM can be considered a disease of the elderly, with median age at diagnosis 65 years, and incidence of GBM in patients aged ≥65 years rising rapidly 10.

Patients with good performance status are typically treated with 60 Gy of standard long‐course radiotherapy (LCRT) over 6 weeks with conventional fraction sizes of 1.8–2.0 Gy, with concurrent TMZ 4. However, the optimal treatment strategy for older patients remains controversial. Although the addition of RT, compared to best supportive care, may be associated with better survival outcomes and equivalent quality of life outcomes in the elderly, the optimal RT dose fractionation and the benefit of chemotherapy in this population is an area of active investigation 11, 12, 13, 14, 15, 16, 17, 18, 19, 20, 21, 22.

A randomized controlled trial (RCT) and several retrospective studies conducted in the elderly suggest that short course radiation therapy (SCRT) of 34–40 Gy in 2.6–3.4 Gy fractions, with or without TMZ, may have similar results to LCRT 23, 24, 25. Results from the Nordic trial suggested that SCRT may be superior to LCRT in patients aged ≥70 years 26. Based on the available data, SCRT is recommended in the National Comprehensive Cancer Network (NCCN) guidelines as a Category 1 treatment option for patients >70 years old with Karnofsky performance status (KPS) of ≥60 or <60 27.

Given the evolving story on RT fractionation for elderly patients with GBM, we used the National Cancer Data Base (NCDB), a national patient registry, to determine RT treatment trends in the United States, identify demographic, clinical, and treatment factors associated with the use of SCRT, and evaluate survival outcomes for elderly GBM patients ≥70 years old treated with SCRT versus LCRT.

## Materials and Methods

### Study population

We queried the National Cancer Data Base (NCDB), a hospital‐based prospective patient registry of American College of Surgeons Commission on Cancer accredited facilities, which captures approximately 70% of all cancers diagnosed in the United States, to identify elderly patients, defined as ≥70 years‐old at the time of diagnosis, who were diagnosed with GBM between 1998 and 2011. Patients with radiation dose fractionation details were included, and divided into two groups based on receipt of either SCRT (34–42 Gy in 2.5–3.4 Gy fractions), or LCRT (58–63 Gy in 1.8–2.0 Gy fractions). The full exclusion criteria are summarized in Table 1.

**Table 1:** Exclusion criteria

Exclusions	Number of patients excluded	Number of patients remaining
Total number of glioblastoma multiforme cases, 1998–2011	—	114,979
History of cancer	13,709	101,270
Metastatic disease	620	100,650
Vital status or follow‐up data missing	7782	92,868
Missing treatment information	12,210	80,658
Treatment other than surgery, radiation, or chemotherapy	325	80,333
Received chemotherapy only	446	79,887
Received surgery with or without chemotherapy only	16,828	63,059
Treatment classified as palliative care	1498	61,561
Radiation therapy preceded surgery	2342	59,219
Histology: WHO grade I–III	859	58,360
No surgery and no biopsy or missing biopsy data	1459	56,901
Missing radiation dose	17,020	39,881
Missing number of radiation treatments	3951	35,930
Radiotherapy technique other than external beam^a^	546	35,384
Total dose other than 58–63 Gy or 34–42 Gy	10,478	24,906
Dose/Fraction other than 1.8–2.0 Gy to 58–63 Gy or 2.5–3.4 Gy to 34–42 Gy	3682	21,224
Age <70 years	16,626	4598
Final study population	—	4598

SCRT, hypofractionated short‐course radiotherapy; LCRT, long‐course radiotherapy using conventional fractionation.

a Other radiotherapy modalities such as radiosurgery, brachytherapy, and Gamma Knife were excluded.

### Study variables

Demographic data on age, gender, race (white, black, or other), insurance status (private, Medicare, Medicaid/other government insurance, or uninsured), median income (<$30,000, $30,000–$35,999, $36,000–$45,999, or ≥$46,000 as determined by zip code), education level (percent of the population without a high school degree as determined by zip code), and year of diagnosis were collected. KPS and Charlson–Deyo comorbidity index scores were extracted to control for underlying patient health status. Treatment facility type was collected (categorized as community programs, comprehensive community programs, or academic/research institutions). Treatment details were extracted including extent of resection, and receipt of chemotherapy.

### Statistical methods

The primary outcome of interest was overall survival (OS). The non‐parametric Wilcoxon–Mann–Whitney test was used to assess difference in median age while Chi‐square tests were performed to analyze difference in distribution of categorical variables. Survival rates were estimated using the Kaplan–Meier method, and log‐rank tests were used to determine statistical significance. Crude and adjusted hazard ratios (HR) with 95% confidence intervals (CI) were calculated using Cox regression modeling. Pre‐selected covariates were assessed with descriptive statistics, and included in the multivariable model if significantly associated with RT dose fractionation scheme. All tests were two‐sided; *P* < 0.05 were considered statistically significant. Statistical computations were performed on SAS 9.3 system (SAS Institute, Cary, NC) or GraphPad prism software (version 3.0, GraphPad Software).

## Results

### Study population and treatment patterns

**Figure 1:**
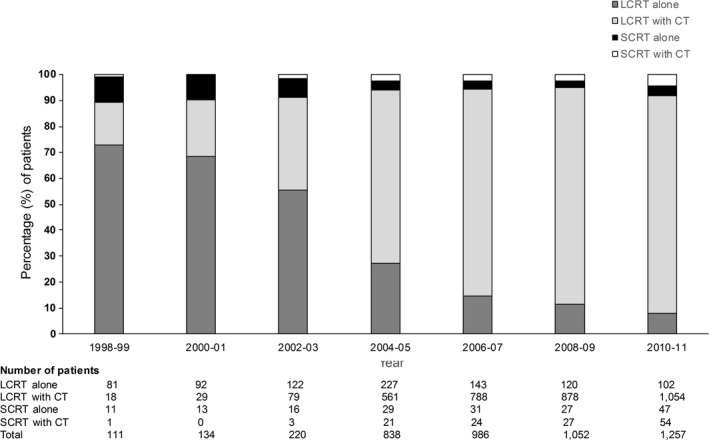
Radiation fractionation and chemotherapy treatment patterns by year of diagnosis for elderly patients aged ≥70 years with glioblastoma multiforme in the National Cancer Database, 1998–2011. SCRT, hypofractionated short‐course radiotherapy; LCRT, long‐course radiotherapy using conventional, fractionation, CT, chemotherapy.

A total of 4598 patients were identified in the NCDB database who met our inclusion and exclusion criteria (Table 1). Of the patients who met our selection criteria, 304 (6.6%) underwent SCRT and 4294 (93.4%) underwent LCRT. Between 1998 and 2011, the percentage of patients receiving SCRT ranged from 5.1% in 2008–2009 to 10.8% in 1998–1999 (Fig. 1). The majority of patients (*n* = 3537; 76.9%) received chemotherapy. The percentage of elderly patients who received chemotherapy rose from 17.1% in 1998–1999 to 88.1% in 2010–2011 (Fig. 1).

### Factors associated with SCRT

Patients who received SCRT tended to be older than patients who received LCRT, with a median age of 78 versus 75 years (*P* < 0.0001; Table 2). SCRT patients were more likely to be female (53.0%) than LCRT patients (44.6%, *P* = 0.005). In terms of race, 91.8% of patients who received SCRT were white, compared to 94.6% of patients who received LCRT (*P* = 0.011). There was no significant difference comparing the two fractionation groups with respect to insurance status, median income, or education level. SCRT patients had higher Charlson–Deyo comorbidity scores compared to the LCRT group, with 16.9% versus 10.8% having a comorbidity score of ≥2, respectively (*P* = 0.006). KPS performance status was only available for 3.1% of our patient population (*N* = 141), of whom 83% (*N* = 117) had scores of ≥60. Almost half of patients who underwent SCRT (49.2%) were treated at academic/research institutions compared to 37.2% of patients who received LCRT (*P* = 0.0001). SCRT patients were more likely to receive biopsy only without tumor resection compared to LCRT patients (34.5% vs. 19.5%, *P* < 0.0001), and were less likely to receive chemotherapy compared to LCRT patients (42.8% vs. 79.3%, *P* < 0.0001).

**Table 2:** Patient and treatment characteristics by radiotherapy dose fractionation

	All patients (*N* = 4598)	LCRT (*N* = 4294)	SCRT (*N* = 304)	*P*
Age (years); Median (IQR)	75 (7)	75 (6)	78 (9)	<0.0001
Gender *N* (%)				0.005
Males	2523 (54.9)	2380 (55.4)	143 (47.0)	
Females	2075 (45.1)	1914 (44.6)	161 (53.0)	
Race *N* (%)				0.011
White	4339 (94.4)	4060 (94.6)	279 (91.8)	
Black	149 (3.2)	139 (3.2)	10 (3.3)	
Other	110 (2.4)	95 (2.2)	15 (4.9)	
Insurance status *N* (%)				0.065
Private	505 (11.1)	477 (11.3)	28 (9.3)	
Medicare	3949 (87.0)	3685 (87.0)	264 (87.4)	
Medicaid/other government	70 (1.5)	60 (1.4)	10 (3.3)	
Uninsured	15 (0.33)	15 (0.35)	0 (0.0)	
Median income *N* (%)				0.084
<$30,000	459 (10.5)	423 (10.3)	36 (12.4)	
$30,000–$35,999	788 (18.0)	745 (18.2)	43 (14.8)	
$36,000–$45,999	1161 (26.5)	1096 (26.8)	65 (22.4)	
≥$46,000	1979 (45.1)	1833 (44.7)	146 (50.3)	
Education level *N* (%)				0.177
≥29%	548 (12.5)	509 (12.4)	39 (13.5)	
20–28.9%	927 (21.1)	863 (21.1)	64 (22.1)	
14–19.9%	1100 (25.1)	1043 (25.5)	57 (19.7)	
<14%	1812 (41.3)	1682 (41.1)	130 (44.8)	
Year of diagnosis *N* (%)				0.031
1998–2004	944 (20.5)	801 (18.7)	72 (23.7)	
2005–2001	3654 (79.5)	3493 (81.4)	232 (76.3)	
Charlson–Deyo score *N* (%)				0.006
0	3015 (70.3)	2831 (70.5)	184 (67.7)	
1	794 (18.5)	752 (18.7)	42 (15.4)	
≥2	479 (11.2)	433 (10.8)	46 (16.9)	
Facility type *N* (%)				0.0001
Community program (CP)	311 (6.8)	298 (7.0)	13 (4.3)	
Comprehensive CP	2535 (55.2)	2394 (55.9)	141 (46.5)	
Academic/research program	1743 (38.0)	1594 (37.2)	149 (49.2)	
Surgical procedure *N* (%)				<0.0001
Biopsy only	944 (20.5)	839 (19.5)	105 (34.5)	
Tumor resection	3654 (79.5)	3455 (80.5)	199 (65.6)	
Chemotherapy status *N* (%)				<0.0001
No chemotherapy	1061 (23.1)	887 (20.7)	174 (57.2)	
Chemotherapy	3537 (76.9)	3407 (79.3)	130 (42.8)	

SCRT, hypofractionated short‐course radiotherapy; LCRT, long‐course radiotherapy using conventional fractionation; IQR, interquartile range.

Insurance status unknown for *N* = 59; Median income missing for *N* = 211; Education level (percent without a high school degree by zip code) missing for *N* = 211; Charlson–Deyo Score missing for *N* = 310; Facility type missing for *N* = 9.

### Overall survival by RT dose fractionation

**Figure 2:**
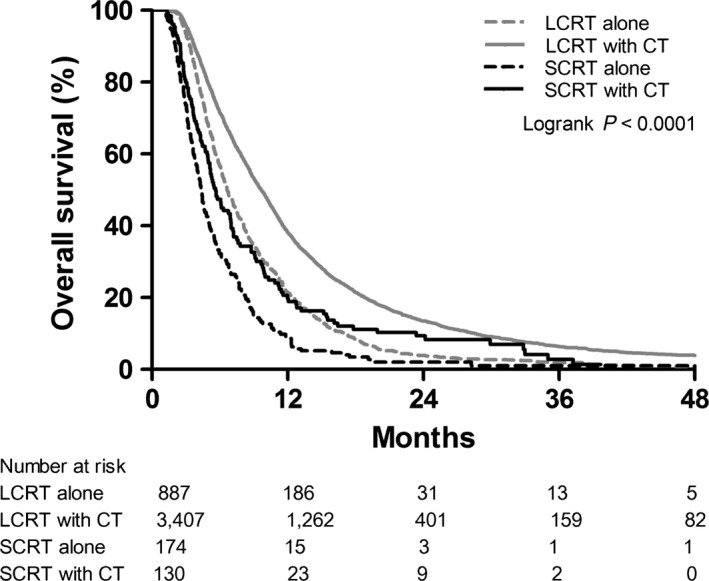
Overall survival by radiation fractionation and receipt of chemotherapy. SCRT, hypofractionated short‐course radiotherapy; LCRT, long‐course radiotherapy using conventional fractionation; CT, chemotherapy.

Median follow‐up was 8.4 months overall; median follow‐up for surviving patients was 21.0 months. Of the 4,598 patients, 4319 deaths were reported. Median OS was 8.6 months for the study group (IQR: 9.6 months), with 1‐year, 2‐year, and 3‐year actuarial survival of 33.3%, 11.0%, and 5.2%, respectively (Table 3). Kaplan–Meier OS estimates are displayed in Figure 2, stratified by RT fractionation and receipt of chemotherapy.

**Table 3:** Overall survival by radiotherapy dose fractionation

	*N*	Events	Median survival in months (IQR)	1‐year actuarial survival (%)	2‐year actuarial survival (%)	3‐year actuarial survival	*P*
Full cohort							
All patients	4598	4319	8.6 (9.6)	33.3	11.0	5.2	N/A
LCRT	4294	4028	8.9 (9.8)	34.7	11.5	5.4	<0.0001
SCRT	304	291	4.9 (5.5)	13.2	5.1	1.8	
No chemotherapy							
All patients	1061	1037	6.3 (6.5)	19.3	3.6	1.7	N/A
LCRT	887	865	6.6 (6.7)	21.5	3.9	1.9	<0.0001
SCRT	174	172	4.3 (4.6)	8.6	2.1	1.0	
Chemotherapy							
All patients	3537	3282	9.5 (10.6)	37.5	13.3	6.2	N/A
LCRT	3407	3163	9.7 (10.8)	38.1	13.5	6.3	<0.0001
SCRT	130	119	5.6 (6.8)	19.7	9.4	2.8	
Biopsy only							
All patients	944	903	5.4 (5.5)	16.1	5.3	2.6	N/A
LCRT	839	802	5.7 (6.0)	17.3	5.6	2.7	<0.0001
SCRT	105	101	3.8 (3.1)	6.1	3.0	2.0	
Tumor resection							
All patients	3654	3416	9.6 (10.3)	37.7	12.5	5.9	N/A
LCRT	3455	3226	9.9 (10.3)	38.9	12.9	6.1	<0.0001
SCRT	199	190	5.6 (6.3)	16.9	6.2	1.8	

IQR, interquartile range; SCRT, hypofractionated short‐course radiotherapy; LCRT, long‐course radiotherapy using conventional fractionation.

Patients who received SCRT had worse OS compared to patients who received LCRT (Table 3), with median survival of 4.9 months (IQR: 5.5 months) versus 8.9 months (IQR: 9.8 months; *P* < 0.0001). The crude hazard ratio for death comparing SCRT to LCRT was 1.94 [95% confidence interval (CI): 1.71–2.20, *P* < 0.0001]. For SCRT patients, 1‐year, 2‐year, and 3‐year actuarial survival was 13.2%, 5.1%, and 1.8%, respectively, compared to 34.7%, 11.5%, and 5.4% for the LCRT group. Median survival for SCRT patients who received chemotherapy was 5.6 months, compared to 9.7 months for LCRT patients who received chemotherapy (*P* < 0.0001). For patients who did not receive chemotherapy, median survival was 4.3 months for the SCRT group versus 6.6 months for the LCRT group (*P* < 0.0001). Median survival for SCRT patients who received tumor resection was 5.6 months, compared to 9.9 months for LCRT patients who underwent resection (*P* < 0.0001). For patients who underwent biopsy only, median survival was 3.8 months for the SCRT group versus 5.7 months for the LCRT group (*P* < 0.0001).

### Multivariable analysis of overall survival

On multivariable analysis (Table 4), adjusting for age, gender, race, comorbidities, year of diagnosis, facility type, surgery, and chemotherapy, receipt of SCRT remained significantly associated with worse overall survival compared to LCRT, with an adjusted hazard ratio of 1.51 (95% CI: 1.33–1.73, *P* < 0.0001). Age, gender, comorbidities, year of diagnosis, facility type, surgery, and chemotherapy remained significantly associated with overall survival on multivariable analysis (Table 4). The survival detriment with SCRT persisted in a subgroup analysis of patients aged 80 years or older, with an adjusted hazard ratio of 1.31 (95% CI: 1.07–1.62, *P* = 0.011) on multivariable analysis (Table 5).

**Table 4:** Multivariable analysis of overall survival for all patients (age ≥70 years)

	*N*	Events	Crude		Adjusted^a^	
			Hazard ratio (95% CI)	*P*	Hazard ratio (95% CI)	*P*
Age (years)	4282	4005	1.04 (1.03–1.05)	<0.0001	1.03 (1.02–1.04)	<0.0001
Gender						
Male	2347	2210	Ref		Ref	
Female	1935	1795	0.98 (0.92–1.04)	0.418	0.94 (0.88–1.0)	0.040
Race						
White	4041	3784	Ref		Ref	
Black	138	131	1.09 (0.91–1.29)	0.354	1.10 (0.92–1.31)	0.303
Other	103	90	0.86 (0.70–1.06)	0.160	0.80 (0.65–0.99)	0.039
Year of diagnosis						
2003–2004	562	551	Ref		Ref	
2005–2011	3720	3454	0.83 (0.76–0.91)	<0.0001	0.91 (0.83–1.0)	0.050
Charlson–deyo comorbidity score						
0	3013	2822	Ref		Ref	
1	791	731	1.08 (1.0–1.17)	0.065	1.11 (1.02–1.21)	0.012
≥2	478	452	1.28 (1.15–1.41)	<0.0001	1.28 (1.16–1.41)	<0.0001
Facility type						
Community program (CP)	285	275	Ref		Ref	
Comprehensive CP	2335	2190	0.86 (0.76–0.97)	0.016	0.83 (0.73–0.94)	0.003
Academic/research program	1662	1540	0.78 (0.68–0.88)	0.0001	0.76 (0.66–0.86)	<0.0001
Surgical procedure						
Biopsy only	874	833	Ref		Ref	
Tumor resection	3408	3172	0.56 (0.52–0.60)	<0.0001	0.59 (0.54–0.63)	<0.0001
Chemotherapy status						
No chemotherapy	815	793	Ref		Ref	
Chemotherapy	3467	3212	0.61 (0.56–0.65)	<0.0001	0.69 (0.63–074)	<0.0001
Radiotherapy dose fractionation						
LCRT	4011	3747	Ref		Ref	
SCRT	271	258	1.94 (1.71–2.20)	<0.0001	1.51 (1.33–1.73)	<0.0001

SCRT, hypofractionated short–course radiotherapy; LCRT, long–course radiotherapy; using conventional fractionation.

a Multivariable model included all factors with *P* < 0.05 in Table 2 (age, gender, race, Charlson–Deyo comorbidity score, year of diagnosis, facility type, surgery, and chemotherapy). Charlson–Deyo score was not available for 1998–2002; therefore model was restricted to 2003–2011.

**Table 5:** Multivariable analysis of overall survival for patients age ≥80 years

	*N*	Events	Crude		Adjusted^a^	
			Hazard ratio (95% CI)	*P*	Hazard ratio (95% CI)	*P*
LCRT	732	695	Ref		Ref	
SCRT	124	118	1.48 (1.22–1.80)	<0.0001	1.31 (1.07–1.62)	0.011

SCRT, hypofractionated short‐course radiotherapy; LCRT, long‐course radiotherapy using conventional fractionation.

a Multivariate model includes all factors with *P* < 0.05 in Table 2 (age, gender, race, Charlson–Deyo comorbidity score, year of diagnosis, facility type, surgery, and chemotherapy). Charlson–Deyo score was not available for 1998–2002; therefore model was restricted to 2003–2011.

## Discussion

In our large study using a national database of patients aged 70 years or older with glioblastoma multiforme, we found that patients who were treated with hypofractionated SCRT had worse survival compared to patients treated with conventionally‐fractionated LCRT. Patients treated with SCRT tended to be older with more comorbidities, and were less likely to receive resection or chemotherapy. In addition, academic centers were more likely to administer SCRT than community programs. After adjusting for these covariates as well as gender, race and year of diagnosis on multivariable analysis, the survival detriment for patients treated with SCRT compared to LCRT continued to persist. To our knowledge, our report represents the largest study to date comparing survival in elderly GBM patients who received hypofractionated versus conventionally fractionated RT.

Advanced age is a major prognostic factor in GBM, frequently guiding treatment decisions. In addition to potential differences in underlying comorbidities and performance status, younger patients also tend to have a molecularly distinct disease compared to older patients. Specifically, older patients are more likely to show EGFR amplification, loss of 9p, loss of 10q, and gain of chromosome 19 compared to younger patients 28. Current National Comprehensive Cancer Network (NCCN) guidelines offer treatment recommendations for patients with GBM based on age (>70 years vs. ≤70 years) 27. In this study, patients who received SCRT were significantly older than patients who received LCRT (median age 78 years vs. 75 years), explaining in part the worse survival associated with hypofractionation.

The major limitation of our study is that KPS performance status was only available for 3.1% of the patient population (*N* = 141). Of note, 83% of the 141 patients with documented KPS (*N* = 117) were recorded as having scores of 60 or higher. Without performance status for the majority of patients, we accounted for patients’ underlying health status by adjusting for comorbidities as measured by the Charlson–Deyo comorbidity index. In addition to age and comorbidities, it is certainly probable that physicians were more likely to recommend SCRT for patients with worse performance status, such that the association between SCRT and worse overall survival was confounded by performance status.

Our finding that SCRT is associated with worse survival compared to LCRT differs from the results of several published randomized trials. The non‐inferiority randomized controlled trial (RCT) of patients aged ≥60 years by Roa et al. accrued 100 patients between 1996 and 2001. It was published in 2004 and found similar overall survival comparing LCRT to SCRT (40 Gy in 15 fractions), at 5.1 months versus 5.6 months, respectively (*P* = 0.57) 23. KPS was not significantly different in the two arms. However, only 72% of the LCRT patients completed their radiation treatment, compared to 95% of the patients in the SCRT arm; in addition, the trial was not powered to detect an absolute difference in survival and patients did not receive chemotherapy. In our study, all included patients completed their radiation course, and 43% of SCRT and 79% of LCRT patients received chemotherapy.

In the Nordic RCT of patients aged ≥60 years comparing TMZ alone, LCRT alone, and SCRT alone (34 Gy in 10 fractions), which accrued between 2000 and 2009, a subgroup analysis of patients ≥70 years demonstrated that SCRT was associated with significantly improved overall survival than LCRT [HR 0.59 (95% CI: 0.37–0.93), *P* = 0.02] 26. However, as in Roa et al., a significant number of patients in this study did not complete their course of radiation treatment 23. In addition, it is unclear whether these survival differences would persist in the setting of concurrent and adjuvant chemotherapy.

The use of SCRT was associated with increased receipt of biopsy instead of tumor resection compared to LCRT (35% vs. 20%), accounting in part for the detriment in survival with SCRT given that extent of resection is associated with survival 29. Similarly, less than half of the patients who received SCRT in our study also received chemotherapy. Other prospective and retrospective studies, including a recent analysis of the NCDB database for patients aged ≥65 years diagnosed with GBM between 2005 and 2011, show that patients treated with chemoradiation may have better outcomes than patients treated with chemotherapy or RT alone 12, 30. A smaller NCDB analysis of GBM patients between 2006 and 2011 who received biopsy only also showed that chemoradiation was superior to RT alone 31. In addition, likely reflecting the use of SCRT in the setting of clinical trials, we found that academic centers were more likely to administer SCRT compared to community programs.

In this national database study, median survival for our patients aged ≥70 years was 8.6 months, comparable to results from more recent RCTs in the elderly 11, 14, 26. Survival was highest for elderly patients who received LCRT, resection, and chemotherapy, with median survival approaching 10 months. This figure is still inferior to modern RCTs that included younger patients 5, 6, where median survival is now 16–17 months, and inferior to a Phase II study addressing SCRT with concurrent and adjuvant TMZ in patients ≥70 years‐old with KPS ≥60, where median survival was 12.4 months 21. While prospective studies are likely biased to include healthier patients, compared to the NCDB which captures about 70% of cancer patients treated in the United States, an important question is what the optimal combination of toxic, costly, and time‐consuming treatments including surgery, radiation, and chemotherapy should be for elderly patients with GBM. In our study, of the 4,958 elderly patients treated with radiation with or without surgery and chemotherapy between 1998 and 2011, only 199 (5.4%) were treated with SCRT. Despite multiple RCTs published in the study period 23, 25 that demonstrated similar or even superior survival with SCRT, there did not appear to be a marked increase in the percentage of patients receiving SCRT with time. Future studies will be needed to determine not only the optimal treatment for this patient population, but to address patterns in the adoption of newer treatment paradigms such as SCRT.

There is currently no consistent age threshold for the definition of elderly patients with GBM. In our study, we defined the elderly population as age ≥70 years, in part because the same threshold is used in the NCCN guidelines^27^ and multiple RCTs addressing GBM treatment in the elderly 11, 21, 26, and the median age at diagnosis of GBM is 65 years 10. However, we recognize that as life expectancies continue to improve and cancer is diagnosed at more advanced age, the definition of elderly could shift. We performed a subgroup analysis of patients aged ≥80 years, and although the number of cases diminishes, the survival disadvantage with SCRT remained statistically significant.

Our study has a number of limitations that can be expected with NCDB analyses. The database attempts to capture information on molecular characteristics of the diagnosed cancers but many patients have missing data; for GBM, MGMT methylation status, an important prognostic factor 32, was only available for 2.1% of our patient population (*N* = 98), and 1p19q status was only available for 0.9%. In addition, the granularity between patients receiving gross total resections versus subtotal resections, which has known prognostic importance 29, is not captured by the NCDB. It is possible that patients who received SCRT were less likely to receive gross total resections, which could account in part for poorer survival outcomes in this group. Finally, although we did include receipt of chemotherapy as a variable in our multivariate model, we could not stratify by type of chemotherapy or exact timing of chemotherapy relative to radiation 33, as these details are not captured in the NCDB. Indeed, the patients in our cohort span the era before and after concurrent TMZ became the standard of care 5, 6, 34.

Strengths of our study include the large patient population, which enabled a robust multivariable analysis, and availability of detailed information on radiation dose and fractionation. Furthermore, the study population was likely more representative of the majority of patients seen in our clinics, compared to patients selected for RCTs. Ultimately, the results of our study are hypothesis‐generating and further prospective investigations will be necessary, ideally addressing additional important outcomes in addition to survival including quality of life, financial toxicity, and psychological concerns.

In conclusion, hypofractionated SCRT was associated with worse survival compared to conventionally fractionated LCRT for elderly patients with GBM. Patients treated with SCRT tended to be older with more comorbidities, and were less likely to receive resection or chemotherapy. Outside of a clinical trial where patients are carefully selected and have relatively good performance status, our study presents results from a large national patient registry capturing approximately 70% of GBM patients.

## Conflict of Interest

The authors report no following conflict of interest disclosures.
